# The continuously evolving CRISPR barcoding toolbox

**DOI:** 10.1186/s13059-018-1541-y

**Published:** 2018-09-25

**Authors:** Thomas Gaj, Pablo Perez-Pinera

**Affiliations:** 1 Department of Bioengineering, University of Illinois at Urbana-Champaign, Urbana, IL 61801 USA; 2 Carl R Woese Institute for Genomic Biology, University of Illinois at Urbana-Champaign, Urbana, IL 61801 USA; 3 Carle Illinois College of Medicine, Champaign, IL 61820 USA; 4 Cancer Center at Illinois, University of Illinois at Urbana-Champaign, Urbana, IL 61801 USA

## Abstract

Two articles recently described the development of CRISPR technologies that have the potential to fundamentally transform the barcoding and tracing of mammalian cells.

The adult human body contains over 37 trillion cells, each belonging to one of several hundreds of cell types that have been identified to date [1]; however, this remarkable cellular complexity in combination with current technological limitations is largely responsible for our fundamental lack of understanding of the series of coordinated events that occur during embryonic development—the formation of multicellular organisms from a single primordial cell. Reconstruction of the cellular hierarchies that drive such central functions as organ formation could improve our grasp of many developmental defects and also critically impact our ability to restore or replace morbid tissues.

The rise of cost-effective high-throughput DNA sequencing and the emergence of highly versatile methods for gene editing are now enabling a range of biotechnology applications not previously dreamed possible. Capitalizing on these advances, two groups have now reported the development of technologies that hold the potential to fundamentally transform our knowledge of the molecular and cellular events underlying embryonic development by facilitating accurate tracing of mammalian cells.

In one study, published in *Science*, Kalhor et al. [2] demonstrate the proof-of-principle reconstruction of cell lineages in a mammal, which previously posed significant technical challenges compared with other experimental models, such as zebrafish and reptiles where embryonic development is easier to study [3–5]. The molecular tool that enabled these studies was a self-targeting version of the CRISPR-Cas9 system that relied on a homing guide RNA (hgRNA) which, unlike a normal single guide RNA (sgRNA) consisting of a targeting sequence followed by a scaffold, also encodes a protospacer adjacent motif (PAM) that enables Cas9 to target the expression cassette encoding the hgRNA [6]. As a result of Cas9 self-targeting, stochastic mutations are introduced by non-homologous end joining (NHEJ) repair in the hgRNA-encoding vector, resulting in the generation of a unique barcode that enables tracking of cells in time and space. To demonstrate in vivo cell tracing, Kalhor et al. first created a founder transgenic mouse carrying 41 different hgRNA expression cassettes integrated in the genome, which they named MARC1 (mouse for actively recording cells 1). Subsequently, they induced barcoding by crossing this MARC1 strain with mice that stably express a Cas9 transgene and, at the end point of the study, read the barcodes using high-throughput sequencing.

The authors applied this system to study early lineage segregation in mice and to investigate axis development in the brain, demonstrating that closely related cells have a similar mutation profile, or barcode, unlike those belonging to a different lineage. Overall, the authors created an accurate and robust lineage tree for the early developmental stages in four embryos. However, as acknowledged by the authors, several limitations persist, many of which are inherent to the barcoding system. For example, as a consequence of different hgRNA transcript lengths or integration sites within the genome, the activity of the hgRNAs was found to be variable, which could lead to unpredictability in the generation and analysis of barcodes. Perhaps more limiting was the finding that only a few mutations were detected for each hgRNA, which can be attributed to the NHEJ repair process not generating fully randomized outcomes, but instead introducing only a narrow spectrum of mutations. The authors were able to overcome this problem by studying reads accumulated across multiple barcodes. Based on their experimental data, they concluded that their approach could theoretically generate approximately 10^23^ barcodes by combining reads from ten different hgRNAs, which is sufficient for barcoding each of the approximately 10^10^ cells in a mouse; however, to accomplish this the barcoding system must be refined. One alternative approach that could potentially overcome the shortcomings of this system includes the recently developed EvolvR technology [7].

Arguably, the feature of CRISPR-Cas9 that makes it the most versatile gene-editing platform is its modularity. In its simplest form, a sgRNA guides the Cas9 nuclease to a target site in the genome where it introduces a DNA double-strand break. Importantly, both the sgRNA and the Cas9 nuclease can be re-engineered for improved or even novel capabilities. For example, by introducing two inactivating mutations into Cas9, it can be converted into a mere DNA-binding protein that can then be leveraged to recruit effector domains to target sites. In a study in *Nature*, Halperin et al. [7] used this principle to create EvolvR, a tool to continuously modify all nucleotides within a user-defined genomic window.

The EvolvR systems consists of a Cas9 nickase (nCas9)—a variant of the Cas9 protein that cleaves only one strand of the target DNA sequence—fused to an error prone and nick-translating DNA polymerase, initially a fidelity-reduced variant of DNA polymerase I (PolI) from *Escherichia coli*. Much like other Cas9-based effectors, the nCas9-PolI protein central to EvolvR can be targeted to a specific genomic site using a sgRNA and induce a DNA nick that then stimulates low-fidelity synthesis.

Although simple in design, EvolvR is quite versatile. For example, the authors demonstrated that EvolvR is compatible with different polymerase domains with varying degrees of processivity, which provides an opportunity to customize both the mutagenesis window and mutation rate for specific applications. This is a crucial feature of EvolvR since the editing window can be as large as 350 bp, which in theory can enable more complex and unique randomization than other systems. This is key because an important property of barcoding systems is that they must generate a pool of signatures that are diverse enough to guarantee uniqueness, which for tracing human cells means trillions of barcodes. As indicated above, barcoding systems that rely on the stochastic repair outcome of NHEJ triggered by Cas9-induced double-strand breaks create a limited pool of signatures, a problem that can be overcome by using multiple barcodes per cell, as demonstrated by Kalhor et al. [2]; however, this alternative approach enormously increases the complexity of the computational analysis required to interpret the results. While speculative, it is possible that EvolvR may be used to generate much larger barcode diversity than self-targeting CRISPR-Cas9 systems and simplify the experimental framework by reducing the number of target sites that must be utilized.

Another important feature of EvolvR is that it can capitalize on the multiplexing capabilities of Cas9 to target continuously multiple genomic loci simultaneously. This is particularly important for large-scale evolution of cell function because, by simultaneously interrogating various targets, EvolvR could be used to reprogram whole biosynthetic pathways, a concept first demonstrated as possible through continuous evolution by Wang et al. via the MAGE method which, unlike EvolvR, relied on λ-red-mediated insertion of synthetic DNA fragments into genomic DNA during bacterial replication to facilitate mutagenesis [8].

Although EvolvR was deployed only in bacterial cells, its ability to continuously modify targeted nucleotides holds immense potential for numerous applications in mammalian cells. In fact, while dCas9-mediated recruitment of the activation-induced cytidine deaminase (AID, deaminates cytosine [C] to uracil [U]) can lead to mutagenesis at targeted genomic loci for the creation of complex genetic libraries for protein engineering [9], this system is limited by the breadth of nucleotide substitutions that it can induce. EvolvR, which can introduce each nucleotide at multiple positions, offers an opportunity to survey increased protein sequence space for directed evolution. For instance, the multiplexing capabilities and tunable processivity of EvolvR could be leveraged to generate broadly neutralizing antibodies for therapeutic targets such as HIV, which are known to require improbable mutations. EvolvR could also be used to facilitate the dissection of functional genomic elements. In particular, multiplex homology-directed repair using a complex library of donor templates has been found to be effective at facilitating saturation mutagenesis of a genomic region [10], which in turn can enable quantitative measurements on the effect that a single nucleotide variation can have on factors such as transcript abundance, survival, and function. EvolvR can now potentially provide a means to saturation edit multiple genomic loci simultaneously to, for example, study and perhaps even identify long-range genomic interactions.

However, it remains to been seen whether EvolvR can be deployed in mammalian cells since genotoxicity caused by high mutation rates can adversely impact viability. Nonetheless, the emergence of these and other new technologies are providing researchers with an increasingly sophisticated toolbox that will surely enable mapping and ultimately reconstructing embryonic development. These advances will someday guide not only the restoration or regeneration of defective human tissues but also the creation of improved agricultural systems that can satisfy the continuously evolving demands of our society.

## Abbreviations

hgRNA: Homing guide RNA
NHEJ: Non-homologous end joining
sgRNA: Single guide RNA
